# iNOS Inhibition Reduces Lung Mechanical Alterations and Remodeling Induced by Particulate Matter in Mice

**DOI:** 10.1155/2019/4781528

**Published:** 2019-03-11

**Authors:** Carla Máximo Prado, Renato Fraga Righetti, Fernanda Degobbi Tenorio Quirino dos Santos Lopes, Edna Aparecida Leick, Fernanda Magalhães Arantes-Costa, Francine Maria de Almeida, Paulo Hilário Nascimento Saldiva, Thais Mauad, Iolanda de Fátima Lopes Calvo Tibério, Mílton de Arruda Martins

**Affiliations:** ^1^Department of Bioscience, Federal University of Sao Paulo, Santos, Rua Silva Jardim 136, 11015-020 Santos, SP, Brazil; ^2^Faculdade de Medicina FMUSP, Universidade de São Paulo, Av. Dr. Arnaldo 455, Sala 1210, 01246-903 São Paulo, SP, Brazil; ^3^Hospital Sírio-Libanês, Rua Adma Jafet 115, 01308-060 São Paulo, SP, Brazil

## Abstract

*Background*. The epidemiologic association between pulmonary exposure to ambient particulate matter (PM) and acute lung damage is well known. However, the mechanism involved in the effects of repeated exposures of PM in the lung injury is poorly documented. This study tested the hypotheses that chronic nasal instillation of residual oil fly ash (ROFA) induced not only distal lung and airway inflammation but also remodeling. In addition, we evaluated the effects of inducible nitric oxide inhibition in these responses. For this purpose, airway and lung parenchyma were evaluated by quantitative analysis of collagen and elastic fibers, immunohistochemistry for macrophages, neutrophils, inducible nitric oxide synthase (iNOS), neuronal nitric oxide synthase (nNOS), and alveolar septa 8-iso prostaglandin F2*α* (8-iso-PGF-2*α*) detection. Anesthetized i*n vivo *(airway resistance, elastance, H, G, and Raw) respiratory mechanics were also analyzed. C57BL6 mice received daily 60ul of ROFA (intranasal) for five (ROFA-5d) or fifteen days (ROFA-15d). Controls have received saline (SAL). Part of the animals has received 1400W (SAL+1400W and ROFA-15d+1400W), an iNOS inhibitor, for four days before the end of the protocol. A marked neutrophil and macrophage infiltration and an increase in the iNOS, nNOS, and 8-iso-PGF2 *α* expression was observed in peribronchiolar and alveolar wall both in ROFA-5d and in ROFA-15d groups. There was an increment of the collagen and elastic fibers in alveolar and airway walls in ROFA-15d group. The iNOS inhibition reduced all alterations induced by ROFA, except for the 8-iso-PGF2 *α* expression. In conclusion, repeated particulate matter exposures induce extracellular matrix remodeling of airway and alveolar walls, which could contribute to the pulmonary mechanical changes observed. The mechanism involved is, at least, dependent on the inducible nitric oxide activation.

## 1. Background

In the last few years, special attention has been devoted to the associations between levels of ambient particulate matter (PM) and health effects [1]. It is known that people that live in high polluted places may develop exacerbations of respiratory diseases [2]. However, less is known about the pathophysiology of chronic effects of high PM exposures, although such exposures are also associated with respiratory hospitalizations, including increases in cardiopulmonary mortality and in the rates of lung cancer and in respiratory exacerbations [3, 4].

Significant risks for the development of chronic bronchitis and obstructive airways disease were associated with increased exposure to ambient PM<10 and PM<2.5 *μ*m in diameters (PM10 and PM2.5, respectively) [5]. In addition, Liu et al. [6] demonstrated that PM2.5 exposure decreased small airway function in asthmatic children and inhaled corticosteroid reverted this response improving lung function.

Accordingly, several animal models that mimic susceptible segments of the population are currently being used to elucidate physiological mechanisms [7]. Experimental studies have shown effects of PM exposure to lung function [8, 9]; however, most of them focused in acute effects of PM in the lung. In addition, scarce studies evaluated the effects of air pollution on lung remodeling [10, 11]. Although the extensive studies have evaluated effects of air pollution in health, the mechanisms involved in the lung injury induced by particulate matter were poorly investigated.

Most of the effects of air pollution, including particulate matter exposures, were related to oxidative stress activation [12, 13]. It is also known that a lot inflammatory cascade and modulators released in the inflammatory process are responsible for connective tissue and functional lung alterations observed in an experimental model of chronic lung inflammation and patients with asthma [14–17]. Moreover, is important to note that efficacy of current pharmacological treatments, including inhaled glucocorticoids and cysteinyl leukotriene type 1 receptor antagonists, is partial effective in patients with asthma [18].

Nitric oxide (NO) was associated with a modulation of many physiological effects. NO can be derived either from neuronal nitric oxide synthase (nNOS) and endothelial nitric oxide synthase (eNOS), or from other NO-adduct molecules (nitrosothiols) and it is associated with the airway and vascular tone control. On the other hand, NO derived from inducible nitric oxide synthase (iNOS) seems to be mainly involved in a modulation of immune system [19].

During inflammation, iNOS expression has been described in several types of cells, such as macrophages, neutrophils, and eosinophils, as well as in airway epithelial cells [20, 21]. NO derived from iNOS contributes to airway and distal lung parenchyma response, inflammatory process and extracellular matrix remodeling [22, 23]. Some evidence suggest that nitric oxide can control airway remodeling by interaction with some proteases and antiproteases, such as MMP (metalloproteinase)-12, MMP-9, or growth factors such as TGF (transforming growth factor)-*β* [24, 25], mediators strongly involved in lung remodeling.

Our hypothesis is that repeated PM could induce structural pulmonary changes related to inflammatory response, extracellular matrix remodeling, and oxidative stress activation. In addition, we considered that these alterations are modulated by iNOS activation. Also considering the health effects of air pollution above described, the clinical relevance of this study is related to the possibility that nitric oxide derived from iNOS is involved on lung injury associated with chronic particulate matter exposures since it may represent an important mechanism.

Therefore, our study aims in evaluate the effects of repeated nasal instillations of low dose of residual oil fly ash (ROFA), a concentrate of air pollution resulting from the burning of oil, on pulmonary mechanics and lung inflammation and remodeling. In order to investigate the effects of iNOS activation, we evaluated the effects of the treatment with a highly selective inhibitor of iNOS activity, 1400W, in pulmonary alterations induced by repeated ROFA instillations.

## 2. Methods

All mice received humane care in compliance with the “Guide for Care and Use of Laboratory Animals” (NIH publication 85-23, revised 1985). Animals were housed (12-h light/dark cycle) in plastic cages and received food and water* ad libitum*). All protocols performed in this study were approved by the institutional review board of University of São Paulo (São Paulo, Brazil).

### 2.1. Particulate Material (PM)

Residual oil fly ash (ROFA) was collected from a solid waste incinerator, which is powered by combustible oil, from University Hospital from School of Medicine of University of São Paulo. Characterization of ROFA particles used in this investigation was previously performed [10, 26] by neutron activation to determine the elemental composition, as well as by gas chromatography and high performance liquid chromatography for organics. It was previously determined and detailed described in previous studies [10, 26]. Presence of toxic elements, such as As, Co, Li, and Zn, and several polycyclic aromatic hydrocarbons (PAHs) such as naphthalene, acenaphthylene, fluorene, acenaphthene, anthracene, fluoranthene, pyrene, B[a]anthracene, B[k]fluoranthene, B[a]pyrene, DB[ah]anthracene, B[ghi]perylene, and ind[123cd] were detected and previously detailed [10, 26]. The ROFA used is homogenous in relation to the particles diameter, since more than 80% of the material has particles less than 2.5 *μ*m of diameter [26].

### 2.2. ROFA Instillation

All animals have received 10uL of ROFA solution (concentration of 6mg/mL) daily during the day 1 to 15^th^ by intranasal instillations (Final dose of 60ug/animal/daily) [10]. This dose mimics a mice exposed to 24 hs in a high pollution day of a city as São Paulo, Brazil. Control animals received the same volume of saline intranasal. We did only the control group with 15 days of saline since we have unpublished data showing that five or fifteen instillation of saline did not affect lung inflammation.

### 2.3. 1400W Treatment

To investigate the effects of iNOS inhibition in lung inflammation induced by ROFA instillations, animals were treated for four days with 1400W i.p., in a dose of 2mg/Kg/daily, as previously described [25]. This approach was chosen based in previous studies that have shown that this dose is effective and selective for iNOS and did not cause toxic effects [25, 27]. Control animals were treated with saline solution.

### 2.4. Experimental Design

To investigate the time effects of air pollution in lung, mice received either

(a) intranasal ROFA daily for five days (ROFA-5,* n*=8);

(b) intranasal ROFA daily for fifteen days (ROFA-15,* n*=8);

(c) intranasal saline for fifteen days (SAL,* n*=8);

(d) intranasal ROFA daily for fifteen days and 1400W treatment (ROFA-15+1400W,* n*=8); or

(e) intranasal saline for fifteen days and 1400W treatment (SAL+1400W,* n*=8).

### 2.5. Pulmonary Mechanics Evaluation

Twenty-four hours after the last instillation, the animals were anesthetized with thiopental sodium (80 mg.kg^−1^, i.p.), tracheostomized, and connected to a ventilator to small animals (FlexiVent, SCIREQ, Montreal, Canada). Animals were ventilated at 100 breaths/min with a tidal volume of 20 mL.kg^−1^. This high value of tidal volume is to avoid inefficient ventilation due to an increase in the anatomic death space induced by the inhalation apparatus attached to the ventilator. A dose response curve was performed using 6.125; 12.5; 25; and 50 mg/mL of methacholine inhalation. Methacholine was inhaled for 1 minute and the data was collected 30 sec after the end of inhalation. There was an interval of 3 minutes between the administrations of each dose. The impedance of respiratory system (Zrs) was calculated for each animal and the perturbation volume of 16 sec was used. The same perturbation volume was used after each inhalation. Mechanical ventilation was stopped only to the perturbation application. The cylinder position (Vcyl) and the internal pressure of the cylinder (P cyl) were registered during the 16 sec of perturbation. We used a perturbation of 16 sec, composed by assertions of solenoids with frequencies between 0.25 and 19.625 Hz, avoiding the harmonic distortion [23]. To avoid the lost related to gases, some corrections were performed. Vcyl was correct to obtain a volume that really arrives in the animals (V) and P cyl was correct to the values of Pao, open airways pressure. By the derivation in the time of V, we get the flow (V'). To analyze the impedance, we used the model of constant phase: where the Raw is the airway resistance, Iaw is the inertance, G characterized the energy dissipated to the lung tissue, H characterized the energy accumulated in the lung tissue, and f is the frequency. Results were expressed as percentage of maximal increase (%) after methacholine challenge [23].

### 2.6. Lung Histology

At the end of pulmonary mechanics, the anterior chest wall was opened, animals were exsanguinated via the abdominal aorta and lungs were removed* en bloc*. The lungs were fixed in 10% formaldehyde for 24h in a constant pressure of 20 cmH2O. Sections representing peripheral areas of the lung were cut and processed for paraffin embedding. Five micron sections were stained with Sirius Red collagen and Weigert's technique for elastic fibers quantifications. To analyze collagen and elastic fibers content in airways and lung parenchyma, we measured the total area of airway wall or lung parenchyma tissue, and the collagen or elastic fibers (*μ*m^2^) area in a nine-ten airways or lung parenchyma fields, at a magnification of x10 for airways and x40 for lung parenchyma, in an image analysis system (Image ProPlus 4.6v). Collagen or elastic content (%) in peribronchiolar and alveolar wall was expressed as a relation between the area of collagen or elastic fibers in a specific frame and the total area of the frame [17].

Immunohistochemistry was performed with the following antibodies: Anti-Macrophage-2 (Clone M3/M38, Cedarlane Lab, Burlington, Canada), anti-Neutrophils (MCA771G, Abd Serotec, Oxford, UK), anti-nNOS (nNOS/NOS type I-N31020; BD Transduction Laboratories, San Diego, CA), anti-iNOS (IgG2a–iNOS/NOS type 2–N32020; BD Transduction Laboratories, San Diego, CA) anti-actin (nNOS/NOS type I-N31020; BD Transduction Laboratories, San Diego, CA), anti-8-iso-PGF2*α* (Oxford Biomedical Research, Oxford, UK), goat polyclonal anti-mouse MMP12 (1:200, Santa Cruz Biotechnology, USA), and rabbit polyclonal anti-transforming growth factor-*β*1 (TGF-*β*1) IgG (1:1200, Santa Cruz Biotechnology, USA). Secondary antibodies anti-goat, rat, or rabbit antibodies (Vectastain Abc Kit, Vector Laboratories, USA) were used in accordance with the manufacturer's instructions. Sections were counterstained with hematoxylin.

With a 50-line and 100-point grid connected to the ocular of the microscope, peribronchiolar and alveolar wall density of macrophages, neutrophils, iNOS, and nNOS, TGF-*β* and MMP-12 positive cells were assessed using a point counting technique [28]. Counting was performed in 5 airways (peribronchiolar area) and in 10 parenchyma fields (area of alveolar septa) in each animal at x1,000 magnification by a blinded investigator. The results were expressed as cell/10^4^um^2^. The 8-iso-PGF2*α* area was evaluated by image analysis as described above for collagen and elastic fibers. The 8-iso-PGF2*α*-positive area was expressed as a percentage of the total area in both peribronchiolar and alveolar wall [28].

### 2.7. Statistical Analysis

Statistical analysis was performed using SigmaStat software (SPSS Inc, Chicago, IL). Data were analyzed with One-way Analysis of Variance (ANOVA) followed by the* Holm-Sidak* method for multiple comparisons and data are presented as mean ± SE. To evaluate the effects of iNOS inhibition in ROFA-induced lung alterations, we used Two-way Analysis of Variance, considering one factor the ROFA and the other the 1400W treatment. P<0.05 values were considered significant.

## 3. Results

### 3.1. ROFA Increased the Lung Mechanics Alterations

We noted an increase in the maximal percentage of increase of Rrs and Ers as well as in Gtis and Htis in both ROFA-5 and ROFA-15 compared to saline (P<0.05) as shown in Table 1. There were no significant differences between ROFA-5 and ROFA-15 in all the mechanical parameters.

### 3.2. ROFA Increased Lung Inflammation

ROFA instillations (ROFA-5 and ROFA-15) induced an increase in the number of neutrophils in both peribronchiolar and alveolar wall compared to saline (P<0.05) (Table 2). The number of macrophages was increased only in ROFA-15 in both bronchiolar and alveolar wall compared to saline (P<0.05).

### 3.3. ROFA Increased Oxidative Stress

ROFA instillations (ROFA-5 and ROFA-15) increased the number of iNOS and nNOS-positive cells and the expression of fractional area of 8-iso-PGF2*α* in both peribronchiolar and alveolar wall compared to saline (P<0.05). The expression of 8-iso-PGF2*α* was increased in ROFA-15 group only at airway level (Table 3). Although the iNOS and nNOS-positive cells in airways were increased in ROFA-15 compared to saline group, these values were lower than those obtained in ROFA-5 group (P<0.05). There were no differences in nNOS, iNOS-positive cells and 8-iso-PGF2*α* in alveolar wall between ROFA 5 and ROFA-15 groups.

### 3.4. ROFA Increased Extracellular Matrix Remodeling

Collagen and elastic fibers were increased only in ROFA-15 compared to saline in peribronchiolar and alveolar wall compartments (P<0.001). We also analyzed the number of metalloproteinase 12 (MMP-12) and TGF*β*-positive cells in the lung. There was an increase in TGF*β*-positive cells in both peribronchiolar and alveolar wall in ROFA-5 group and ROFA-15 group compared to saline group (P<0.001). There was a decrease in the expression of TGF-*β* in peribronchiolar wall in ROFA-15d group compared to ROFA-5d group. Considering MMP-12-positive cells, the expression of MMP-12 was higher in AW only in ROFA-15 and in DLP in both ROFA-5 group and ROFA-15 group compared to saline (P<0.001) (Table 4).

### 3.5. Effects of 1400W in the Lung Mechanics Alterations Induced by Fifteen Instillations of ROFA

**Figure 1:**
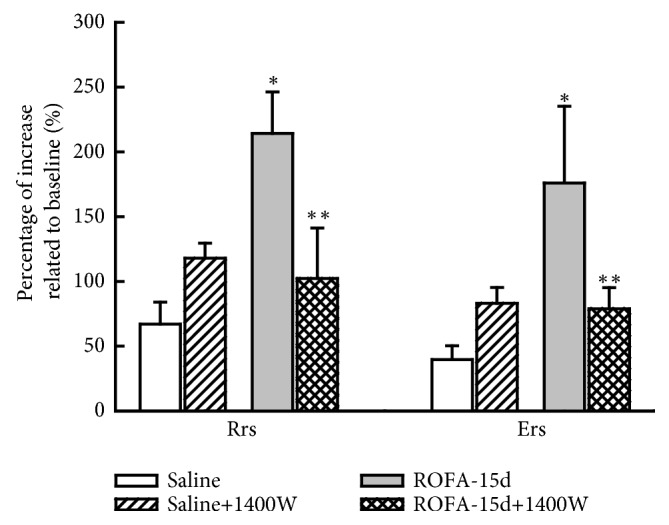
Lung mechanics. This figure represents the mean and SEM percentage of maximal increase of respiratory system resistance (Rrs) and elastance (Ers) relative to baseline. The animals that received fifteen instillations of ROFA presented higher values of both Rrs and Ers compared to control and 1400W treatment reduced this response. *∗p*<0.05 compared with saline groups; *∗∗p*<0.05 compared with animals that received ROFA and vehicle treatment.

The 1400W treatment in animals that received ROFA instillations for 15 days reduced the maximal percentage of increase of Rrs and Ers compared to those received the vehicle (ROFA-15-W compared to ROFA-15d, P<0.05), as shown in Figure 1. There were no significantly effects of 1400W treatment in ROFA-induced an increase in Gtis [ROFA-W: 115.77± 32.22] and Htis [ROFA-W: 77.43± 20.88] values compared to those animals that received ROFA and vehicle (ROFA-15, values of ROFA-15 were shown in Table 1).

### 3.6. Effects of 1400W in the Lung Inflammation Induced by Fifteen Instillations of ROFA

**Figure 2:**
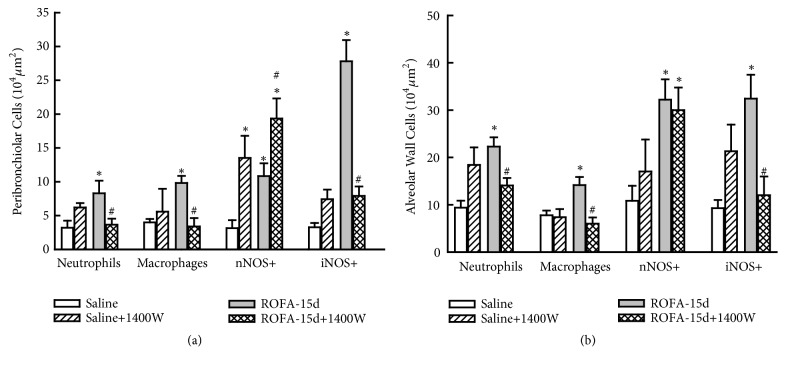
Lung inflammation and nitric oxide synthase expression. This figure represents the mean and SEM values of macrophages, neutrophils, nNOS, and iNOS-positive cells in peribronchiolar (a) and alveolar (b) wall. The animals that received fifteen instillations of ROFA presented higher values of all cells, and 1400W reduced inflammation and iNOS-positive cells, not interfering with nNOS expression. *∗p*<0.05 compared with saline groups; #*p*<0.05 compared with animals that received ROFA and vehicle treatment.

The iNOS inhibition in ROFA-instilled animals (ROFA-15-W) reduced the number of neutrophils and macrophages in both peribronchiolar and alveolar wall compared with those animals that received vehicle (P<0.05) (Figure 2).

### 3.7. Effects of 1400W in the Oxidative Stress Induced by Fifteen Instillations of ROFA

**Figure 3:**
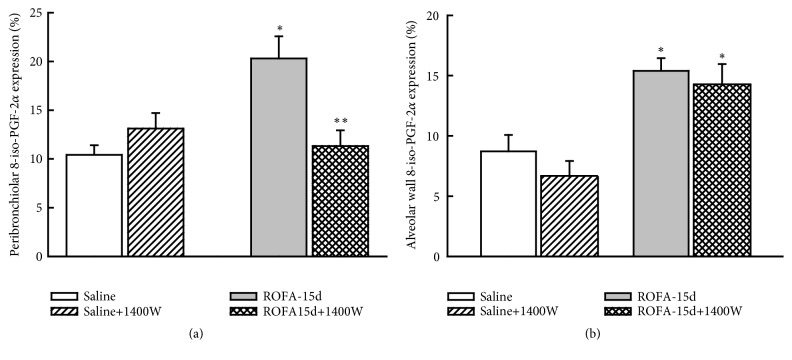
Oxidative stress: this figure represents the mean and SEM values of 8-iso-PGF-2a expression (%) in peribronchiolar and alveolar wall. The ROFA instillations increased the positive area and the iNOS inhibition reduced it only around bronchioles. *∗p*<0.05 compared with saline groups; *∗∗p*<0.05 compared with animals that received ROFA and vehicle treatment.

We observed that in ROFA-15-W there was a decrease in the number of iNOS-positive cells in peribronchiolar and alveolar wall compared to ROFA-15 (P<0.05). As expected, the iNOS inhibition did not affect the nNOS-positive cells in animals that received ROFA-1400W compared to those that received vehicle (Figures 2(a) and 2(b)). The iNOS inhibition reduced the 8-iso-PGF2*α* content in ROFA-15 animals only in airways (P<0.05) (Figure 3).

### 3.8. Effects of 1400W in the Extracellular Matrix Remodeling by Fifteen Instillations of ROFA

**Figure 4:**
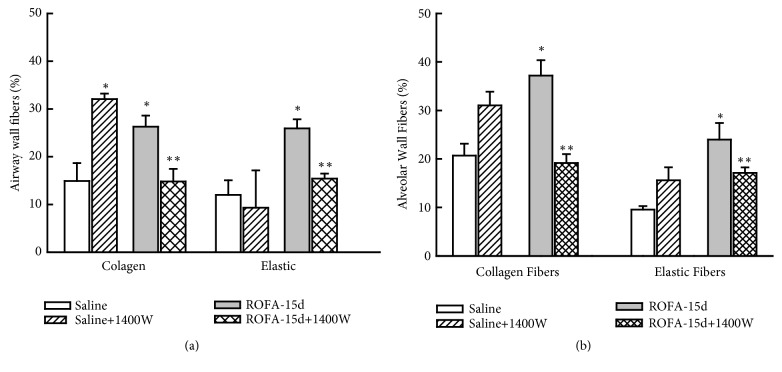
Lung remodeling: this figure represents the mean and SEM values of both collagen and elastic fibers content around airways (a) and in alveolar wall (b). The ROFA instillations increased the airway and parenchyma extracellular matrix fibers deposition compared to control, and the iNOS inhibition reduced these responses. *∗p*<0.05 compared with saline groups; *∗∗p*<0.05 compared with animals that received ROFA and vehicle treatment.

**Figure 5:**
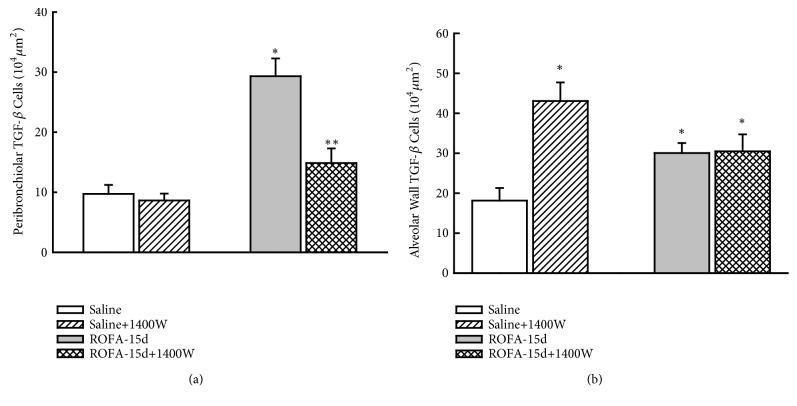
TGF-*β* expression: this figure represents the mean and SEM values of TGF-*β* expression in both peribronchiolar (a) and alveolar (b) wall. The ROFA instillations increased the number of TGF-*β*-positive cells in both airways and alveolar wall and iNOS inhibition reduced this response only in airways. *∗p*<0.05 compared with saline groups; *∗∗p*<0.05 compared with animals that received ROFA and vehicle treatment.

**Figure 6:**
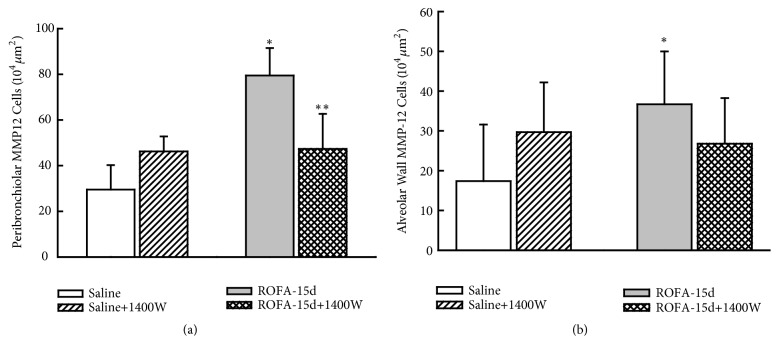
Metalloproteinase 12 expression. This figure represents the mean and SEM values of MMP-12 expression in both peribronchiolar (a) and alveolar (b) wall. The ROFA instillations increased the number of MMP-12-positive cells and iNOS inhibition significantly reduced this response only in airways. *∗p*<0.05 compared with saline groups; *∗∗p*<0.05 compared with animals that received ROFA and vehicle treatment.

Considering pulmonary remodeling (Figure 4), iNOS inhibition reduced both collagen and elastic content in airways and in lung distal parenchyma comparing to those animals that received vehicle (P<0.05). The iNOS inhibition reduced the TGF-*β* (Figure 5) and MMP-12 (Figure 6) positive cells only in peribronchiolar area, not affecting the alveolar wall positive cells (P<0.05).

### 3.9. Qualitative Analysis

**Figure 7:**
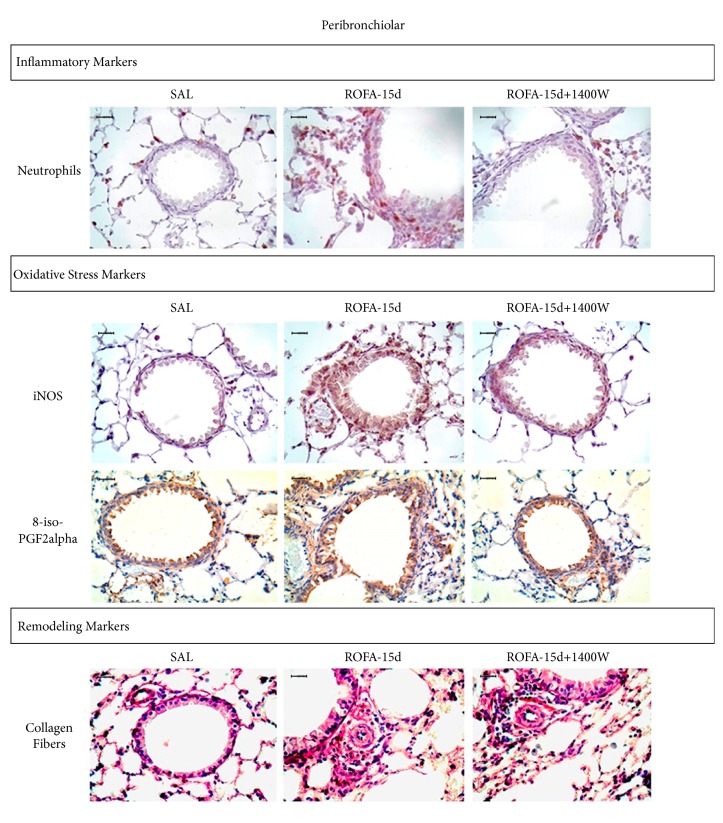
Representative photomicrographs of airway stained with immunohistochemistry to detect neutrophils, iNOS, isoprostane, and collagen fibers in all experimental groups.

Figure 7 shows representative photomicrographs of airway stained with immunohistochemistry to detect neutrophils, iNOS, isoprostane, and collagen fibers in all experimental groups. Peribronchiolar of ROFA-15 animals presented a prominent neutrophil infiltration including iNOS-positive cells and fractional area of the 8-iso-PGF2*α* and collagen fibers. Peribronchiolar sections from animals instilled with ROFA-15 and treated 1400W presented attenuation in neutrophil infiltration including iNOS-positive cells and fractional area of the 8-iso-PGF2*α* and collagen fibers (Scale bar = 30 *μ*m).

## 4. Discussion

In this study we showed that repeated exposures to levels of PM induced significant changes on lung mechanics, lung inflammation, and extracellular matrix remodeling which occurred in both distal airways and lung parenchyma. The inflammatory response was characterized by macrophages and neutrophils and could be observed early after five instillations, while extracellular matrix remodeling associated with increase in MMP-12 and TGF-*β* expression occurred only after fifteen instillations. We also showed that repeated instillations of ROFA induced an increment in the number of nNOS and iNOS-positive inflammatory cells as well as in 8-iso-PGF2*α* expression around peribronchiolar and alveolar wall. This was a feasible and reproducible experimental model for studies of pathophysiological mechanisms involved in lung injury induced by air pollution. The effects of inhaled toxicants and allergen challenge on airway inflammation and oxidative stress has also been studied in well established in vitro experimental animal models [29, 30].

Several pathophysiological mechanisms may be involved in lung injury induced by air pollution [31, 32]. In order to investigate the hypothesis of NO involvement in the pathophysiology of lung inflammation induced by particulate matter, we evaluated the effects of specific iNOS inhibitor in this experimental model. We used 1400W, a highly selective and specific iNOS inhibitor, as previously shown in other experimental studies [25, 27, 33]. The effectiveness of this treatment was assessed by immunohistochemical detection of both nNOS and iNOS. We observed that 1400W treatment only reduced the number of iNOS-positive cells, not affecting the nNOS expression. The most important finding was related to the fact that NO, mainly originated from iNOS activation, was involved in the inflammatory and extracellular matrix remodeling, acting as proinflammatory modulator, since the treatment with 1400W, a high specific inhibitor, reduced the most of the features observed in this model. There were few studies evaluating experimental models of inflammation induced by repeated exposure to air pollution that had mapped the changes induced by chronic exposure of particulate matter not only in the lung parenchyma, but also in the airways [34]. Most of them have combined air pollution exposure to allergic inflammation or tobacco exposure.

It is well know that repeated exposures to irritants such as particulate matter, diesel, cigarette smoke results in bronchial epithelial damage, mucous hypersecretion, fibrosis and narrowing of the airways, destruction of parenchyma and vascular changes [35]. ROFA (residual oil fly ash) is a metal-rich particulate material, with little organic component derived from combustion processes at high temperatures [36]. Due to its high toxicity, it is used as a substitute for experimental environmental studies to assess the biological effects of particulate matter [37–39]. The element composition of ROFA used in the present study was previously characterized [26].

Repeated intranasal instillation of ROFA in mice induced an increased resistance and elastance of the respiratory system as well as the strength and elasticity of lung tissue. Other authors had already demonstrated that ROFA exposures affected bronchial responsiveness. In this sense, Gavett et al. [40] demonstrated that single intratracheal instillation of ROFA in Balb/C altered the total resistance of the respiratory system in response to low doses of methacholine, and induced an accumulation of neutrophils in airways and alveolar walls. Arantes-Costa et al. [10] demonstrated that sensitized animals worsened mucus production when received ROFA after the sensitization period.

The alterations in lung function observed in animals that received ROFA may be secondary to pulmonary morphological alterations found. It is well known that airway extracellular matrix remodeling seen over the years may be associated with the irreversibility of pulmonary function, at least in asthmatic patients [41]. Thus, inflammation and extracellular matrix remodeling observed in ROFA-instilled group may, by itself, lead to increased responses of pulmonary mechanics. Inflammatory cell recruitment is associated with a release of several mediators/modulators, among them nitric oxide, which are also involved in the control of airway smooth muscle contractility and lung parenchyma mechanical responses [16, 25, 42].

In addition, an increased 8-iso-PGF-2*α* in peribronchiolar and alveolar wall could be associated with the responsiveness observed. 8-iso-PGF2*α* concentrations were measurable in exhaled breath condensate (EBC) (Montuschi et al., 2010), a noninvasive technique for sampling airway secretions, and it has been found elevated in patients with COPD compared with healthy ex-smokers [43]. Although previous studies have evaluated the effects of PGE2, which was more potent as a constrictor than PGF2*α*, the latter isoprostane is considered the predominant form generated during free radical attack of cell membranes [44]. Jourdan et al. [45] showed that L-NAME treatment greatly inhibits 8-iso-PGF2*α* and also that pulmonary artery smooth muscle can release this isoprostane. This class of substances induces contractions of the smooth muscle of airways and vessels operating in the Rho tyrosinase and Rho-kinase, leading to increased activity of phosphorylated myosin [16, 22, 46]. Shiraki et al. [47] showed studying tracheal smooth muscle contraction that 8-isoPGF2*α* increased the isometric tension and they concluded that 8-isoPGF2*α* causes airway smooth muscle contraction. However, few studies have evaluated the specific effects of 8-isoPGF2*α* in smooth muscle contraction. Our group also have demonstrated that the inhibition of 8-isoPGF2*α* by different agents reduced the airway hyperresponsiveness [48–50].

Furthermore, collagen and elastic fibers remodeling in the airways and distal lung parenchyma occurred only in ROFA-15 day group. Alterations in the elastic fibers content are known to occur in the pathophysiology of lung disease such as asthma and COPD, a major shortfall of elastic fibers followed by loss of elasticity and elastic recoil occurs in a second stage regeneration of these fibers, making them thicker. It would be expected that an increase in neutrophilic recruitment may reduce the elastic fiber content. However, corroborating our results, other authors have found an increase in elastic fibers [42, 49], probably because of the turnover processes of elastic fibers that involves the function of smooth muscle promoting elastin formation and the elastolytic effects of inflammation, particularly related to neutrophils, which was prominent in both peribronchiolar and alveolar wall in the present study.

In an attempt to understand some of the mechanisms involved in these responses, we evaluated the 8-iso-PGF-2*α* expression in the lung, a marker of oxidative stress pathway activation. The formation of peroxynitrite leads to lipid peroxidation and generation of isoprostanes such as 8-iso-PGF2 *α*, which is one of the predominant forms generated during the attack of free radicals in the cell membrane [50]. After both 5 and 15 days of ROFA exposures there was an increase in the oxidative stress pathway activation in the lung and an increase in the number of nNOS and iNOS-positive inflammatory cells in peribronchiolar and alveolar wall. As NO has a short half-life, the reactive oxygen (ROS) and reactive nitrogen species (RNS) can act as NO carriers, culminating with oxidative stress activation.

In addition, it is well known that NO modulates bronchial smooth muscle contractility and also lung parenchyma. In this regard, 1400W treated animals had a significant reduction in the resistance and elastance of the respiratory system compared to vehicle treated groups, suggesting an effect of this inhibitor attenuating the response of lung mechanics induced by ROFA in proximal and distal airways. However, there were no differences in Htis and Gtis, which reflects more lung distal parenchyma response.

Donors of nitric oxide (nitrovasodilator) are relaxing the smooth muscles, including airway smooth muscle [51, 52]. The effects of NO as a bronchodilator is well recognized, however various studies emphasized that the effects of NO on bronchodilation depends on the enzyme type that is producing NO. When NO was produced by iNOS in high quantities, it could induce bronchoconstriction [24]. Besides the direct effects of NO in smooth muscle is important to note that the control of inflammation and remodeling by the treatment of iNOS inhibition can also contribute to the improvement of lung function.

We found that iNOS inhibition reduced the infiltration of neutrophils and macrophages and deposition of collagen and elastic fibers. This effect was similar in airways and lung parenchyma. Some mechanisms may explain how nitric oxide acts on inflammatory cells. High levels of NO act as effectors molecule of the immune system and can inhibit DNA synthesis by inactivation of ribonucleic reductase and by direct deamination of DNA [24]. The activation of apoptosis can be induced by nitric oxide since it can influence DNA fragmentation and prolong cell survival [53]. However, Chung et al. [54] suggest that NO may have effects, both apoptotic and antiapoptotic, and that these responses depend on nitric oxide levels and also the producing cell.

Corroborating the present findings, we recently showed that iNOS inhibitor attenuates lung vascular remodeling in an asthma model [24]. Some evidence tried to explain how NO affect the collagen deposition. Horstman et al. [55] suggested that nitric oxide may attenuate vascular remodeling secondary to hypoxia in rats. Aristoteles et al. [25] studied a model of chronic allergic inflammation and demonstrated that nitric oxide inhibition derived from the inducible isoform modulates the extracellular matrix remodeling.

Other possible explanations are the role of nitric oxide in the modulation of metalloproteinases synthesis and activation as well as in their inhibitors [56]. Metalloproteinases are families of enzymes that are involved in the degradation of collagen and other extracellular matrix proteins [16].

We evaluated the MMP-12 since this metalloproteinase is able to degrade extracellular matrix components and is involved in lung tissue remodeling observed in inflammatory pulmonary diseases such as chronic obstructive pulmonary diseases (COPD). We observed that the number of MMP-12-positive cells was increased in both peribronchiolar and alveolar wall after fifteen ROFA instillations. Corroborating the idea that nitric oxide influences metalloproteinases, we observed that 1400W treatment reduced the number of MMP-12 positive cells only around peribronchiolar area, suggesting that iNOS is involved in MMP-12 upregulation induced by particulate matter, and it could be one of the mechanisms in which iNOS acts in lung remodeling. These results are in accordance with previous studies [17, 27, 57].

Another important mechanism involved in lung remodeling is the profibrotic cytokine TGF- *β*. It is produced by a number of cells, including macrophages, epithelial cells, and fibroblasts and is involved in most of the cellular biological processes of airway remodeling [58]. In the present study, we observed that both five and fifteen instillations of ROFA induce an increase in both peribronchiolar and alveolar wall TGF- *β*.-positive cells. The iNOS inhibition reduced this response only in peribronchiolar cells, not affecting alveolar wall.

1400W treatment reduces the 8-iso-PGF-2*α* only around airways, suggesting that oxidative stress is one important mechanism involved in air pollution-induced lung alterations, as extensively discussed in the literature [59–61]. Other studies of our group have shown this same effect of iNOS inhibition in oxidative stress using an animal model of chronic allergic lung inflammation [62, 63].

The major limitation of the present study was related to the fact that ROFA is a tool to study the effects of particulate matter in lung and may be not representative of particulate matter present in air pollution. However, its exact composition is known and makes the experiments reproducible which is important to elucidate mechanisms involved. The clinical relevance is related to two important views: First, we developed an experimental model to study air pollution with repeated instillations that can be used to investigate physiopathological mechanisms. Second, we clearly showed that iNOS is involved in lung inflammation induced by particulate matter. Further studies involved other mechanics such as the role of oxidative stress, IL-17 and cholinergic anti-inflammatory system, as recent studies suggested [64].

Collectively, ROFA instillation induced lung mechanics, inflammatory, and extracellular matrix remodeling alterations in lungs of C57BL mice. These changes are associated with an increased in the oxidative stress pathway, metalloproteinase, and TGF-*β* activation. NO, derived mainly from iNOS, is involved in proinflammatory and profibrotic alterations induced by particulate matter.

## Figures and Tables

**Table 1:** *ROFA instillation-induced lung mechanics alterations*: after both five (ROFA-5d) and fifteen instillations (ROFA-15d), mice presented an increase in the percentage of respiratory system resistance (%Rrs) and elastance (%Ers) as well as in lung tissue impedance (%Htis) and lung tissue resistance (%Gtis) compared to control that received only saline. *∗*p<0.05 compared to saline (SAL group).

GROUPS	%Rrs	%Ers	%Gtis	%Htis
*SALINE*	67.05±16.99	39.70±10.51	40.36±6.65	33.23±7.47
*ROFA-5d*	213.58±29.06*∗*	149.62±42.57*∗*	124.93±20.72*∗*	74.72±10.26*∗*
*ROFA-15d*	214.18±31.97*∗*	175.95±59.21*∗*	106.56±42.25*∗*	104.99±12.56*∗*

**Table 2:** *ROFA-induced lung inflammation*: after both five (ROFA-5d) and fifteen instillations (ROFA-15d), mice presented an increase in the number of neutrophils and inflammatory cells that express nNOS and iNOS around airways and in distal lung parenchyma. The macrophages were significantly increased only in ROFA-15d groups compared to saline. *∗*P<0.05 compared to saline.

	Airway		Distal lung parenchyma	
	Macrophage	Neutrophils	Macrophage	Neutrophils
SAL	3.9±0.5	3.2±1.1	7.8±0.98	9.4±1.5

ROFA-5d	10.7±6.5	8.3±1.4*∗*	8.3±1.4	18.9±1.8*∗*

ROFA-15d	9.0±1.0*∗*	8.3±1.9*∗*	14.16±1.71*∗*	22.3±1.9*∗*

**Table 3:** *ROFA-induced oxidative stress*: after both five (ROFA-5d) and fifteen instillations (ROFA-15d), mice presented an increase in the number of neutrophils and inflammatory cells that express nNOS and iNOS around airways and in distal lung parenchyma. The macrophages were significantly increased only in ROFA-15d groups compared to saline. *∗*P<0.05 compared to saline; #P<0.05 compared to ROFA-5d.

	Airway			Distal lung parenchyma		
	iNOS	nNOS	8-iso-PGF2*α*	iNOS	nNOS	8-iso-PGF2*α*
SAL	3.3±0.6	3.13±1.20	10.42±0.98	9.30±1.70	10.80±3.20	8.71±1.36

ROFA-5d	44.5±5.0*∗*	20.01±3.65*∗*	8.73±1.51	33.0±7.4*∗*	33,40±5.50*∗*	13.53±0.87*∗*

ROFA-15d	27.82±3.14*∗*,#	10.83±1.9*∗*#	20.30±1.51*∗*	32.43±5.04*∗*	32.40±5.00*∗*	15.39±1.05*∗*

**Table 4:** *ROFA-induced extracellular matrix remodeling*: after both five (ROFA-5d) and fifteen instillations (ROFA-15d), mice presented an increase in the number of neutrophils and inflammatory cells that express nNOS and iNOS around airways and in distal lung parenchyma. The macrophages were significantly increased only in ROFA-15d groups compared to saline. *∗*P<0.05 compared to saline; *∗∗*P<0.05 compared to ROFA-5d.

	Airway				Distal lung parenchyma			
	Collagen fibers	Elastic fibers	MMP-12	TGF-*β*	Collagen fibers	Elastic fibers	MMP-12	TGF-*β*
SAL	15.7±2.9	13.5±1.6	29.5±4.4	9.7±1.5	20.7±2.5	9.5±0.7	17.4±5.4	18.1±3.2

ROFA-5d	13.9±3.2	11.1±1.7	46.6±17.4*∗*	34.6±3.7*∗*	20.9±2.6	17.74±1.6	55.7±10.1*∗*	46.3±4.5*∗*,

ROFA-15d	26.7±1.9*∗*, *∗∗*	25.4±2.4*∗*, *∗∗*	79.5±5.3*∗*	37.2±3.2*∗*	37.2±3.2*∗*, *∗∗*	23.9±3.4*∗*	36.7±4.4*∗*	30.1±2.5*∗*, *∗∗*

## Data Availability

The data used to support the findings of this study are available from the corresponding author upon request.
